# Comparison of growth characteristics of *in vitro* cultured granulosa cells from geese follicles at different developmental stages

**DOI:** 10.1042/BSR20171361

**Published:** 2018-04-27

**Authors:** Yan. Deng, Xiang. Gan, Da. Chen, Hulian. Huang, Junsong. Yuan, Jiamin. Qiu, Shenqiang. Hu, Jiwei. Hu, Jiwen. Wang

**Affiliations:** Farm Animal Genetic Resources Exploration and Innovation Key Laboratory of Sichuan Province, Sichuan Agricultural University, Chengdu, Sichuan 611130, P.R. China

**Keywords:** Cell culture, Geese, Granulosa cells, Growing characteristics, Gene expression

## Abstract

Granulosa cells (GCs) are essential components of follicles and are involved in regulating the process of follicles development. However, comparative studies on GCs isolated from different staged follicles have not been conducted in goose. The aim of the present study was to identify the growth characteristics of goose GCs from pre-hierarchical (6–10 mm) and hierarchical (F4–F2, F1) follicles. Our results showed that the three cohorts of cells had different tolerance to collagenase and had noticeable morphological differences. The F1 granulosa layers were fully digested by 0.1% collagenase, while higher concentration (0.3%) was used for both F4–F2 and pre-hierarchical granulosa layers. In the state of suspension, the diameter of F1 individual cell was larger than the other two cohorts. However, after adhering to the culture plate, cells of F1 just had changes in the diameter accompanied by small bright spots, while both pre-hierarchical and F4–F2 GCs proliferated rapidly with spreading and irregularly shaped voids. Furthermore, all attached cells could be stained by the follicle-stimulating hormone receptor antibody. Analyses of both growth curve and the mRNA expression profiles of genes related to cellular proliferation, apoptosis, and steroidogenesis suggested that three cohorts of *in vitro* cultured GCs had different physiological viability and functions. Taken together, the present study not only revealed differences of the growth characteristics among three cohorts of goose GCs from pre-hierarchical, F4–F2 and F1 follicles, but also optimized the *in vitro* culture system of geese different staged GCs.

## Introduction

Granulosa cells (GCs) are the key follicular somatic compartments surrounding the oocytes and play an essential role in maintaining normal ovarian functions [1,2]. GCs are involved in a broad range of cellular physiological processes during the development of follicles, including mechanical support, nutrient intake [3,4], theca cell differentiation [5], and steroidogenesis [6,7]. In particular, its own proliferation, differentiation or apoptosis has a determinative effect on the fate of developing follicles [8].

In laying chickens, ovarian follicles are usually arranged in a strict follicular hierarchy where follicles are mainly divided into pre-hierarchical (<8 mm in diameter) and hierarchical (>9 mm in diameter; designated as F5–F1, F5 < F4 < F3 < F2 < F1) follicles [9]. During the development of follicles, the granulosa layer increases in its cell number and surface area, and changes simultaneously occur in the thickness and number of granulosa layers [10,11]. Meanwhile, GCs go through the morphological change from flattened to cuboidal cells with follicular maturation [11]. In addition, the process of follicular development is closely associated with the degree of GCs differentiation. In general, GCs are arrested in an undifferentiated state during the pre-hierarchical phase but become differentiated when entering into the hierarchy [12,13]. Notably, the steroidogenic capacity of GCs also varies with follicular growth and development, with the maximum in secreting progesterone (P4) in the largest hierarchical follicle (F1) [14]. Pawlowska et al. [15] have shown that levels of steroidogenic acute regulatory protein (*StAR*) and cholesterol side chain cleavage (*CYP11A1*) mRNAs remained constantly low in pre-hierarchical GCs but increased gradually in the hierarchy with the maturation of follicles. Similarly, very low levels of 3β-hydroxysteroid dehydrogenase (*3β-HSD*) mRNA were observed in GCs of pre-hierarchical follicles in contrast with its highest levels in the F1 GCs [14,15]. Notably, GCs prior to follicle selection are more susceptible to undergoing apoptosis compared with those of hierarchical follicles [8,16,17]. Collectively, these data reveal morphological and functional differences among GCs of follicles at different developmental stages.

In order to better understand the molecular and cellular mechanisms controlling avian follicular development, it is of importance to establish *in vitro* culture models of GCs from follicles at different stages of development. Although some studies have successfully isolated and cultured GCs from either pre- or hierarchical follicles [9,18–25], a comparative study on the growing characteristics of *in vitro* cultured GCs isolated from different sized follicles as well as the optimization of each culture condition is still required. The objectives of the present study were to first optimize the *in vitro* culture condition of each cohort of goose GCs (namely pre-hierarchical (6–10 mm), F4–F2, and F1 GCs respectively) including the concentrations of both collagenase and fetal bovine serum (FBS), and then to compare their growing characteristics during *in vitro* culture through morphological observation, growing curve and investigation of expression of genes related to cellular proliferation, apoptosis, and steroidogenesis.

## Materials and methods

### Animals and isolation of GCs

The healthy maternal line of Tianfu meat geese (*Anser cygnoides*, 35–45 weeks) during the period of egg laying were used in the present study. The geese were kept under natural light and temperature condition at the Waterfowl Breeding Experimental Farm of Sichuan Agricultural University (Sichuan, China), and were provided with unlimited access to feed and water. Individual laying cycles were monitored for each goose throughout the laying sequence. Geese were killed by cervical dislocation 7–9 h before oviposition.

Follicles from each ovary were dissected and subsequently washed with ice-cold sterile phosphate buffered saline (PBS, pH 7.4. Solarbio), and morphologically normal follicles were characterized based on a previously described report [26]. The granulosa layers from both F4–F2 and F1 follicles were isolated according to the method introduced by Gilbert et al. [18]. Briefly, a cut (about 1 cm long) was made with a scalpel blade along the stigma of follicles, and then the follicular compartments were inverted into a dish containing PBS. Next, the residual yolk was washed out and granulosa layers were collected. As to the pre-hierarchical follicles, follicles were first made a cut with a scalpel blade. Next, two crooked tweezers were utilized to clamp the incision and then to split the follicular walls gently. Then, a tweezer was used to clamp one incision and follicular walls were washed with PBS until granulosa layers were peeled off from the follicular walls. After collecting the granulosa layers from different sized follicles, they were cut into pieces and transferred into 15 ml of centrifuge tubes, respectively, and 0.1% or 0.3% type II collagenase (Sigma) was used to disperse those granulosa layers. During the digestion, the tubes were shaken at 37°C in a water bath for no more than 3 min, until the granulosa layers were fully dispersed. Dulbecco modified Eagle medium/nutrient and F-12/1:1 (DMEM/F12, HyClone) was utilized to dilute the concentration of collagenase. The dispersed cells were finally filtered through a 200-mesh sieve and centrifuged for 10 min at 1000 rpm. Both freshly dispersed and suspended cells were captured under a microscope (Olympus, Tokyo, Japan) and the latter’s diameter was measured using the OPTPro software. All procedures using birds in the present study were carried out according to the Guide of the Faculty Animal Care and Use Committee, Sichuan Agricultural University, Sichuan, China.

### Culture of GCs

The GCs were harvested from pre-hierarchical (6–10 mm), F4–F2, and F1 follicles. The culture medium used in the study consisted of DMEM/F12, 10% FBS (Gibco), 1% streptomycin, and penicillin mixture (Gibco). The cells resuspended with above medium were seeded on different culture plates and then incubated at 37°C under 5% CO_2_ in humidified air to allow the cells to reach a confluence. After incubation of around 6 h, the medium was replaced by fresh medium. The six-well plates (∼15 × 10^5^ cells/well) were used for cell immunofluorescence assay, and the 12-well plates (∼10 × 10^5^ cells/well) were used for qPCR. In addition, the 96-well plates (∼5 × 10^5^ cells/well) were used for the collection of images and the growth curve of GCs. For three groups of GCs cultured *in vitro*, each experiment was conducted in triplicate.

### Morphological observation and cell viability assay

Before analysis of cell viability, the morphology of GCs was observed per 24 h for a total period of 168 h, and the field was randomly selected to capture using a microscope (Olympus, Tokyo, Japan). Cell viability was analyzed using the MTT [3-(4,5-dimethylthiazol-2-yl)-2,5-diphenyltetrazolium bromide] (Amresco) method. Briefly, the cells were treated with 0.5–1 mg/ml MTT for additional 4 h at different time points (24, 48, 72, 96, 120, 144, and 168 h), with ten replicates and two negative controls for each cohort at each point. Then, MTT was removed and DMSO (Solarbio) was inserted with 150 µl/well, and the plate was shaken for 10 min [27]. The absorbance of cells at 490 nm was measured using an automatic enzyme immunoassay analyzer.

### Immunofluorescence assay

Follicle-stimulating hormone receptor (FSHR) immunostaining was used to identify GCs from different sized follicles after 72 h of incubation, since FSHR is exclusively present in avian GCs [28]. In brief, each well was washed three times with PBS to remove the culture medium. Then, the GCs were fixed with 4% paraformaldehyde (Solarbio) for 30 min, and penetrated with 0.1% Triton-X (Amresco) for 15 min. After that, the GCs were blocked with 4% BSA (Solarbio) for 1 h and washed with PBS. The rabbit-anti-goose-FSHR primary antibody (diluted 1:200 with 1% BSA; Bioss) was then added to the wells and incubated overnight at 4°C. After that, the cells were washed three times with PBS and incubated with a FITC-goat anti-rabbit IgG antibody (diluted 1:500 with 1% BSA; Boster) at 37°C for 2 h. Finally, the nuclei was labeled with DAPI (4,6-diamidino-2-phenylindole; 10 µg/ml in PBS; BiYunTian Biotechnology) (Solarbio) for 10 min and images were taken using a florescence microscope (Nikon). For three groups of GCs cultured *in vitro*, each experiment was conducted in triplicate.

### RNA extraction and real-time PCR

According to the manufacturer’s protocol, total RNA was extracted using TRIzol reagent (Invitrogen), and the quality was assessed by spectrophotometric absorbance at 260/280 nm. First-strand cDNA was synthesized from 1 µg of total RNA using a cDNA synthesis kit (TaKaRa) following the manufacturer’s instructions. Reactions of quantitative real-time PCR (qPCR) were performed in a CFX96^TM^ Real-Time system (Bio-Rad, CA, U.S.A.) using the SYBR PrimerScript^TM^ real-time PCR kit (TaKaRa). The procedure included 1 cycle of 95°C for 30 s, followed by 40 cycles of 95°C for 5 s, and primer-specific annealing temperature for 30 s. An 80-cycle melting curve was performed, starting at a temperature of 85°C and increasing by 0.5 every 10 s to determine primer specificity. Only one product of the desired size was identified and one single peak was observed in a melting curve for each primer. Each sample was repeated three times and the relative mRNA expression of genes was normalized to *β-actin* and *GAPDH* using the 2^− ΔΔ*C*^_t_ method [29]. The primers designed for qPCR are shown in Table 1.

**Table 1:** Primer pairs for real-time quantitative PCR

Gene	Sequence (5′-3′)	*T*_m_ (°C)	Size (bp)
*3β-HSD*	F: GACCTGGGGTTTGGAATTGAG	60	170
	R: TAGGAGAAGGTGAATGGGGTGT		
*StAR*	F: AGAATCTTGACCTCTTTGACGCTG	60	87
	R: GAGACGGTGGTGGATAACGGA		
*CYP11A1*	F: AGGGAGAAGTTGGGTGTCTACGA	60	89
	R:CGTAGGGCTTGTTGCGGTAGT		
*Caspase-3*	F: CTGGTATTGAGGCAGACAGTGG	62	158
	R: CAGCACCCTACACAGAGACTGAA		
*Bcl-2*	F:GATGCCTTCGTGGAGTTGTATG	60	100
	R: GCTCCCACCAGAACCAAAC		
*CCND2*	F: TTCATCGCCCTTTGTGCC	60	80
	R: ATTGCTCCCACGCTTCCA		
*p21*	F: TGAGGCAACACCTGGAAGAAG	60	207
	R: CCTTAGATGGGACCTTGTGGG		
^1^*GAPDH*	F: TTTCCCCACAGCCTTAGCA	60	90
	R: GCCATCACAGCCACACAGA		
^1^*β-Actin*	F: CAACGAGCGGTTCAGGTGT	59.6	92
	R: TGGAGTTGAAGGTGGTCTCG		

F: sense primers; R: antisense primers.

1 Housekeeping gene for data normalization.

### Data analysis

Analyses of data from cell diameter, cell viability, and the relative genes mRNA expression levels were subjected to ANOVA, and the means were assessed for significant differences using the Tukey’s test. All results were expressed as mean ± S.D. and a *P*-values below 0.05 was considered statistically significant. All statistical analyses were carried out using SAS 8.1 (SAS Institute, Cary NC, U.S.A.).

## Results

### Morphological differences of GCs

**Figure 1:**
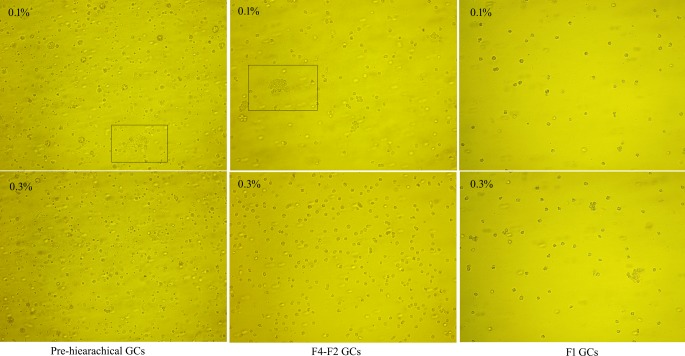
Digestion of GCs from follicles at different developmental stages The concentrations of collagenase are 0.1% and 0.3%, and the morphological differences among three cohorts of GCs cultured *in vitro* were observed under a microscope (×100). The rectangular box indicates the granulosa layer without being fully digested. “F1 GCs” represents the GCs from the largest follicle (F1) and “F4–F2 GCs” represents the GCs from the fourth to the second largest follicles (F4–F2).

Three cohorts of granulosa layers were isolated from different sized follicles according to the methods mentioned in the “Materials and methods” section. Although 0.1% and 0.3% type II collagenase were able to fully disperse the F1 granulosa layers, 0.3% collagenase led to cell loss and decreased cell viability during digestion (Figure 1). In contrast, the pre-hierarchical and F4–F2 granulosa layers could only be fully dispersed by 0.3% collagenase which did not impair cell viability (Figure 1). Thus, we finally used 0.1% collagenase to digest the F1 granulosa layers while 0.3% for the pre-hierarchical and F4–F2 granulosa layers.

**Figure 2:**
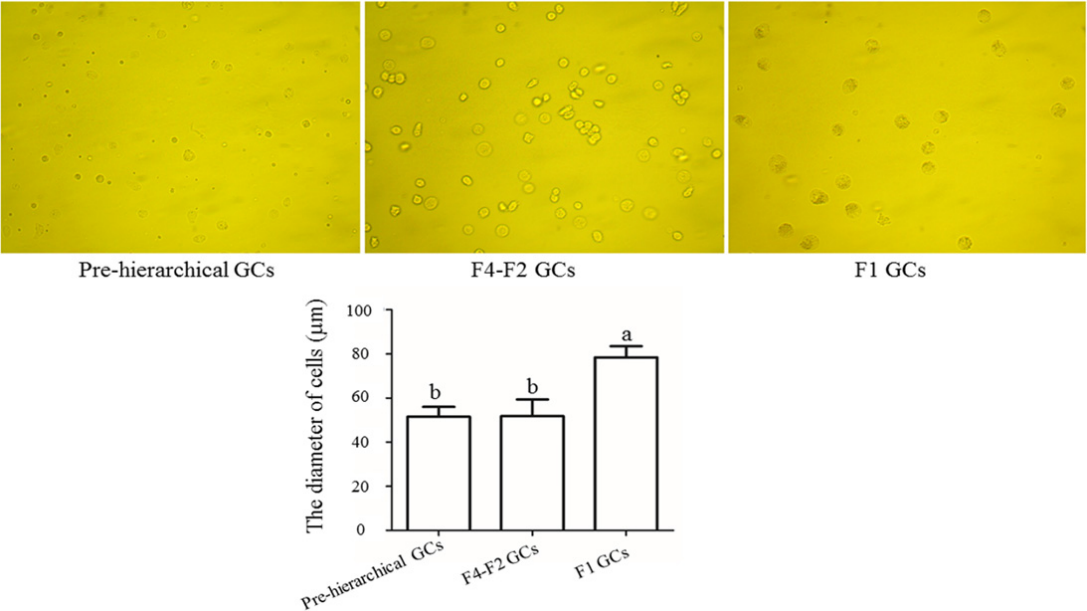
Differences in the diameter of freshly suspended GCs from follicles at different developmental stages Morphological differences among three cohorts of GCs cultured *in vitro* were observed under a microscope (×400). The diameter of three cohorts of GCs is displayed below and different lowercase letters indicate significant differences at *P*<0.05. “F1 GCs” represents the GCs from the largest follicle (F1) and “F4–F2 GCs” represents the GCs from the fourth to the second largest follicles (F4–F2).

Microscopic examination showed that the freshly suspended cells presented either round or oval shapes. Noticeably, the diameter of F1 GCs was larger than that of the other two cohorts of GCs (Figure 2, *P*<0.05).

**Figure 3:**
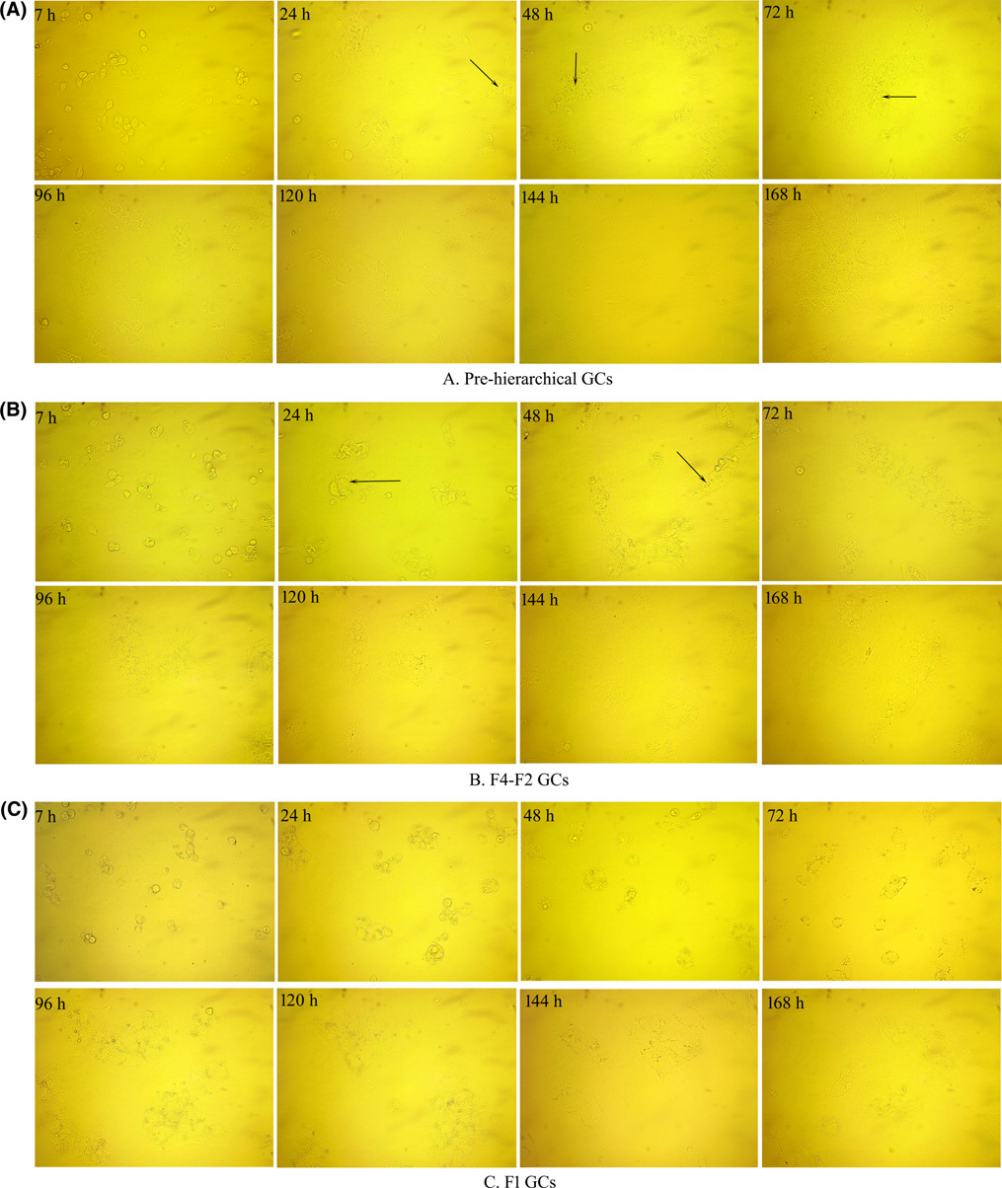
Morphological differences among three cohorts of adhered GCs Changes in the morphology of three cohorts of GCs during the *in vitro* culture period from 7 to 168 h were observed under a microscope (×400). Arrows indicate the irregularly shaped voids. “A/B/C” represents the GCs of pre-hierarchical, F4–F2, and F1 follicles respectively. “F1 GCs” represents the GCs from the largest follicle (F1) and “F4–F2 GCs” represents the GCs from the fourth to the second largest follicles (F4–F2).

As shown in Figure 3, all cultured cells adhered to the bottom of plates and were shown as roundness or oval after 7 h of incubation (Figure 3A–C). Pre-hierarchical GCs spread more rapidly than the other GCs and displayed filamentous extensions at 24 h of culture (Figure 3A). Irregularly shaped voids were observed in both F4–F2 and pre-hierarchical GCs (Figure 3A,B; 24 h), but F1 GCs only increased in cell size (Figure 3C; 24 h). During the incubation from 48 to 72 h, those irregular voids seen in pre-hierarchical and F4–F2 GCs became smaller but their number increased gradually, and the cells from the two stages proliferated significantly (Figure 3A,B; 48–72 h) while F1 GCs still changes in cell size (Figure 3C; 48–72 h). At 96 h of culture, F1 GCs began to fuse by spreading all over the bottom of plates (Figure 3C), while the pre-hierarchical and F4–F2 GCs increased in cellular density although their layers became thinner with increasing time of culture (Figure 3A,B; 96 h). When cultured to 120 h, the F1 GCs started to be senescent (Figure 3C), while the adhered cell layers of F4–F2 and pre-hierarchical GCs were extremely thin and grew over each other (Figure 3A,B). In addition, numerous tiny bright spots were observed in F1 GCs (Figure 3C; 24–120 h) but not in F4–F2 and pre-hierarchical GCs (Figure 3A,B).

### Growth activity of GCs cultured *in vitro*

**Figure 4:**
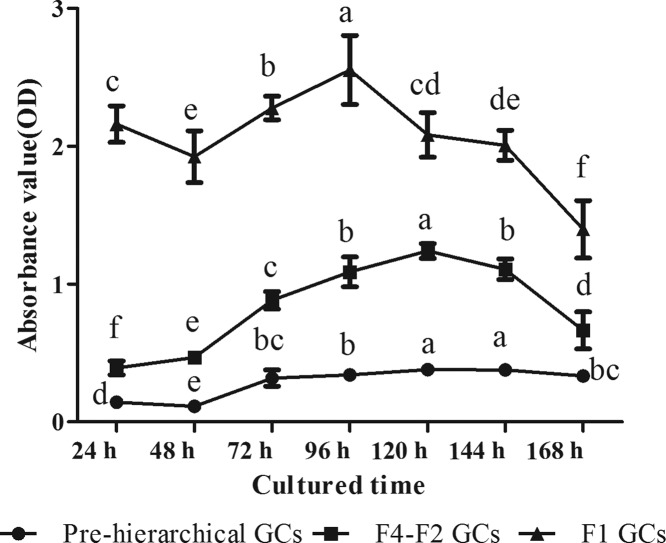
Growth curve of three cohorts of *in vitro* cultured GCs Different lowercase letters indicate significant differences at *P*<0.05 among different culture time within the same cohort of cells. “F1 GCs” represents the GCs from the largest follicle (F1) and “F4–F2 GCs” represents the GCs from the fourth to the second largest follicles (F4–F2).

Growth curve showed that all GCs entered the logarithmic phase at 48 h of culture, and it went on for ~48 h for F1 GCs and 72 h for pre-hierarchical and F4–F2 GCs (Figure 4). During this period, cell activity increased fast and significantly (Figure 3A,B; 48–120 h; Figure 3C; 48–96 h) (*P*<0.05). However, activity of F1 GCs decreased gradually at 120 h while that of either F4–F2 or pre-hierarchical GCs decreased at 144 h of culture (Figure 4).

### Identification of GCs using FSHR immunostaining

**Figure 5:**
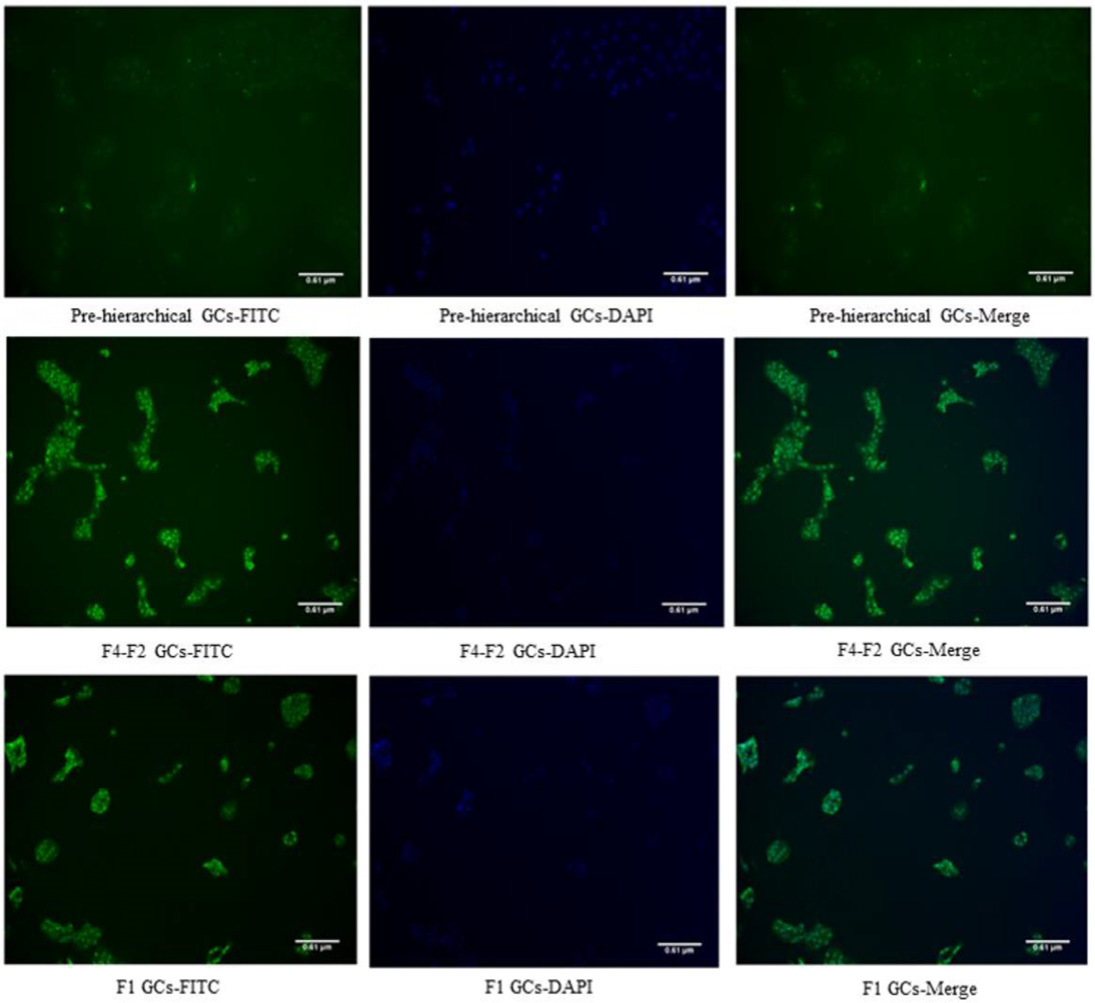
Immunofluorescence identification of three cohorts of GCs using the FSHR antibody “F1 GCs” represents the GCs from the largest follicle (F1) and “F4–F2 GCs” represents the GCs from the fourth to the second largest follicles (F4–F2).

The cultured GCs at 72 h were used for immunofluorescence assay, and a FSHR primary antibody (rabbit-anti-goose) was utilized to identify GCs. Our results showed that all GCs were marked with a green outline (Figure 5), indicating that there was no other cell contamination.

### Expression patterns of several genes related to basic functions of GCs during *in vitro* culture

**Figure 6:**
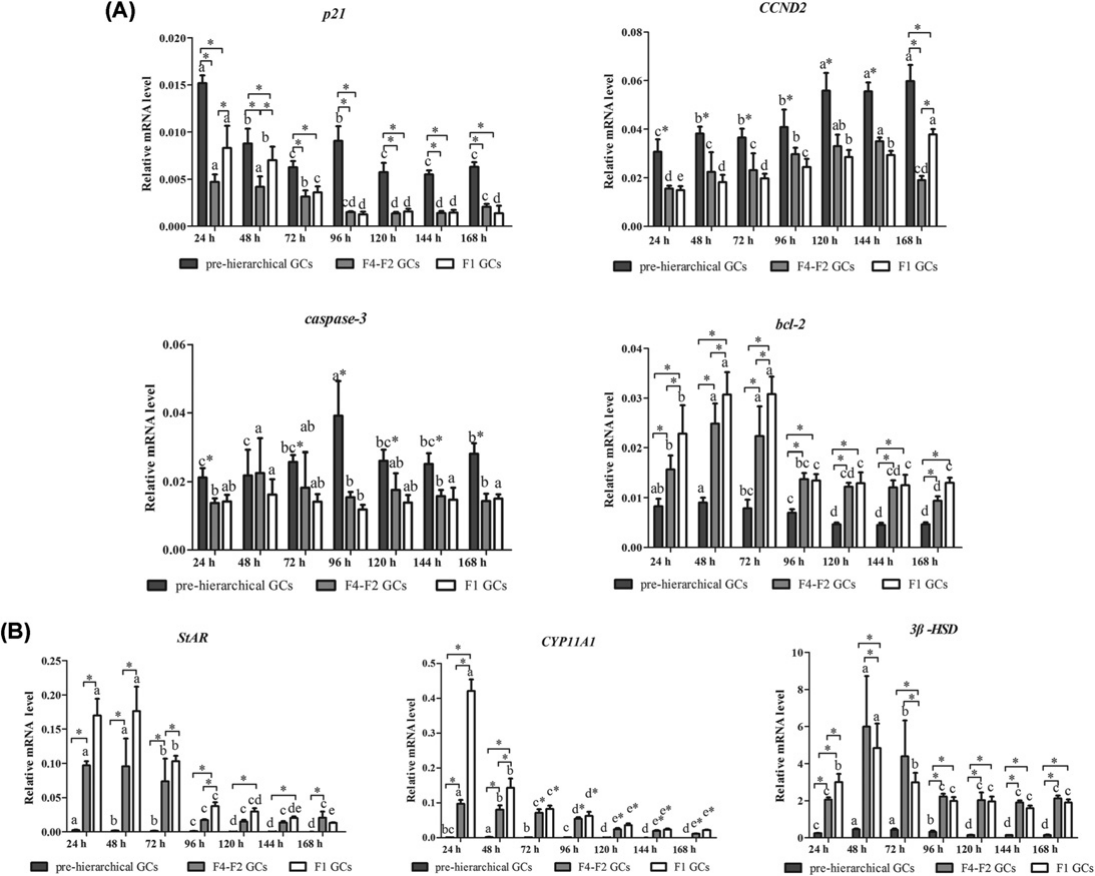
Expression patterns of several genes related to basic functions of GCs from different sized follicles during *in vitro* culture (A) and (B) represent expression of proliferation, apoptosis genes, and expression of steroidogenesis genes mRNA respectively. Bars with different lowercase latter are significantly different for the same type of cell during the cultured time *in vitro* (*P*<0.05). * indicates significant differences among GCs of three different stages in the same cultured time (*P*<0.05 and *P*<0.01 respectively). *GAPDH* and *β-actin* were used as internal controls. “F1 GCs” represents the GCs from the largest follicle (F1) and “F4–F2 GCs” represents the GCs from the fourth to the second largest follicles (F4–F2).

Our results showed that the expression levels of genes related to proliferation and apoptosis including Cyclin D2 *(CCND2), p21*, and *caspase-3* were significantly higher in pre-hierarchical than in hierarchical GCs (*P*<0.05), and there was no significant difference between F1 and F4–F2 GCs (*P*>0.05). However, expression of *p21* in F1 GCs was higher than in F4–F2 GCs (*P*<0.05). In addition, levels of *bcl-2* (anti-apoptotic) were markedly higher in hierarchical than in pre-hierarchical GCs (*P*<0.05). For either F1 or F4–F2 GCs, *bcl-2* mRNA levels increased significantly during the incubation period from 24 to 72 h (*P*<0.05), followed by an abrupt decline at 96 h of culture, and then remained statistically unchanged (*P*>0.05). The mRNA levels of *p*21 gradually decreased with increasing time of culture while *CCND2* presented an opposite trend. As for *bcl-2* and *caspase-3*, their expression showed a tendency of increasing early and decreasing later during incubation (Figure 6A).

In addition, the expression levels of several steroidogenic genes including *StAR, CYP11A1*, and *3β-HSD* were higher in hierarchical than in pre-hierarchical GCs during *in vitro* culture (*P*<0.05, Figure 6B). The pre-hierarchical GCs had lower expression levels of *StAR* and *CYP11A1* but relatively higher levels of *3β-HSD*. Besides, expression of steroidogenic genes was significantly higher in F1 than in F4–F2 GCs at 24 h of culture (*P*<0.05, Figure 6B). As the cells were cultured from 48 to 72 h, expression of *3β-HSD* was significantly higher in F4–F2 than in F1 GCs (*P*<0.05, Figure 6B), while expression of *StAR* and *CYP11A1* reached the maximum in F1 GCs (*P*<0.05, Figure 6B). After that, there was no significant difference in the levels of those genes between F1 and F4–F2 GCs (*P*>0.05). With increasing time of culture, expression of *StAR* and *CYP11A1* gradually decreased, while that of *3β-HSD* increased first and then decreased in each cohort of GCs (Figure 6B).

## Discussion

In the avian ovary, GCs play essential roles during the processes of follicular recruitment, selection, maturation, and ovulation. Previous researchers have used 0.1% collagenase to disperse the F3–F1 granulosa layers or 0.3% collagenase for the F4–F2 granulosa layers in chicken [9,23]. Based on previous reports [9,19–25], in the present study we further improved the methods used for isolation and culture of GCs from geese follicles at different developmental stages through optimizing the concentrations of collagenase and FBS. The results showed that the F1 granulosa layers were fully dispersed by 0.1% collagenase, but a much higher concentration (0.3%) of collagenase was required to digest the granulosa layers of both pre-hierarchical and F4–F2 follicles (Figure 1).

Gilbert et al. [30] have shown that changes in the diameter of granulosa cells were dependent on follicular size, which was also related to corresponding changes in the cell shape. With regard to the freshly dispersed GCs, we found that the diameter of F1 cells was significantly larger than that of either pre-hierarchical or F4–F2 ones (*P*<0.05, Figure 2). During the incubation from 7 to 72 h, the F1 GCs only increased in their diameter, however, GCs of pre-hierarchical and F4–F2 follicles spread more rapidly until fusion, coupled with the formation of filamentous extensions and irregularly shaped voids. After that, the F1 cells began to attach and fuse together, while the pre-hierarchical and F4–F2 GCs continued proliferating with their cell layers becoming much thinner (Figure 3A–C). The growth curve displayed that all groups of GCs reached the logarithmic phase at 48 h of culture, and it lasted 48 and 72 h for the F1 and the other GCs respectively (Figure 4). Thereafter, the cells came into the plateau stage within the incubation of 120–144 h, followed by the senescence phase (Figure 4). These results altogether suggested that the three populations of geese GCs showed both similar and different growth characteristics during *in vitro* culture, which was to a larger extent consistent with the phenomenon seen in other cell types [31]. Additionally, the identification of isolated geese GCs were verified by IF assay with the specific FSHR primary antibody, showing that no other cell contaminations were present in all three cohorts of GCs (Figure 5). Thus, it was concluded that the three populations of geese GCs were successfully isolated and cultured under our optimal condition and exhibited different growth characteristics during *in vitro* culture.

It has been well recognized that changes in the morphology of GCs were closely related to some biological processes such as cell proliferation, apoptosis, and steroidogenesis [32–34]. To further explore differences in the basic functions of the three cohorts of GCs, expression of several key genes associated with cell proliferation and apoptosis was first determined. Among these genes, *CCND2* and *p21* were recognized as their roles in controlling cell cycle by stimulating and inhibiting cell proliferation respectively [35,36], while *bcl-2* and *caspase-3* function as an anti- and pro-apoptotic gene respectively [16,17]. In the present study, results from the expression of *caspase-3* and *bcl-2* further demonstrated that the pre-hierarchical GCs were much more susceptible to apoptosis than the hierarchical ones, which were in accordance with others’ reports [16,17,37,38]. Besides, the pre-hierarchical GCs also exhibited a high proliferation rate, as demonstrated by both the expression of *CCND2* and *p21* (Figure 6A) and the observed morphological changes during *in vitro* culture (Figure 3A). Since the mRNA expression of *bcl-2* reached its maximum in the F1 GCs, indicating that these GCs were much more resistant to apoptosis. In contrast with *CCND2*, the mRNA expression levels of *p21* were higher in F1 than F4–F2 GCs, which indicated that F4–F2 GCs proliferated more quickly than F1 ones, which were also demonstrated by their morphological changes (Figure 3C). Additionally, the mRNA expression of key steroidogenic genes (e.g. *CYP11A1, StAR*, and *3β-HSD*) [14,39–42] were also investigated to further define the steroidogenic characteristics of GCs from different sized follicles. The results showed that the expression of *CYP11A1* and *StAR* in the F1 GCs was highest at 72 h of culture, but was extremely low in pre-hierarchical ones during *in vitro* culture. However, expression of *3β-HSD*, a key gene controlling P4 production [14,39], reached the maximum in the F4–F2 GCs but was relatively lower in pre-hierarchical ones when cultured to 72 h. These results were consistent with previous studies [14,40,43], suggesting that large amounts of P4 were mainly produced in hierarchical follicles with the maximum in the F1 GCs, followed by the F4–F2 GCs, whereas the pre-hierarchical GCs were not able to produce P4. Taken together, differences observed in the expression patterns of key genes related to proliferation, apoptosis, and steroidogenesis during *in vitro* culture further supported the notion that there were significant differences in both morphological and functional characteristics among geese GCs isolated from different sized follicles.

In conclusion, the present study not only established an optimal culture condition for each cohort of GCs isolated from geese follicles at different developmental stages, but also revealed the differences in their morphological and functional characteristics during *in vitro* culture. These data may contribute to a better understanding of the mechanisms controlling avian follicle growth and development.

## Supporting information

## Abbreviations

*3β-HSD*: 3β-hydroxysteroid dehydrogenase
FSHR: follicle-stimulating hormone receptor
GC: granulosa cell
