# Supplementary material for: Comparison of growth characteristics of in vitro cultured granulosa cells from geese follicles at different developmental stages
Source: Biosci Rep. 2018 Apr 27;38(2):BSR20171361. doi: 10.1042/BSR20171361 (PMC5920135; doi:10.1042/BSR20171361)
Supplement: Supplementary file 1 [file bsr20171361_Supp1.pdf]

The original pictures from 96 h to 168 h for pre-hierarchical, F4-F2 and F1 GCs:

Pre-hierarchical GCs:

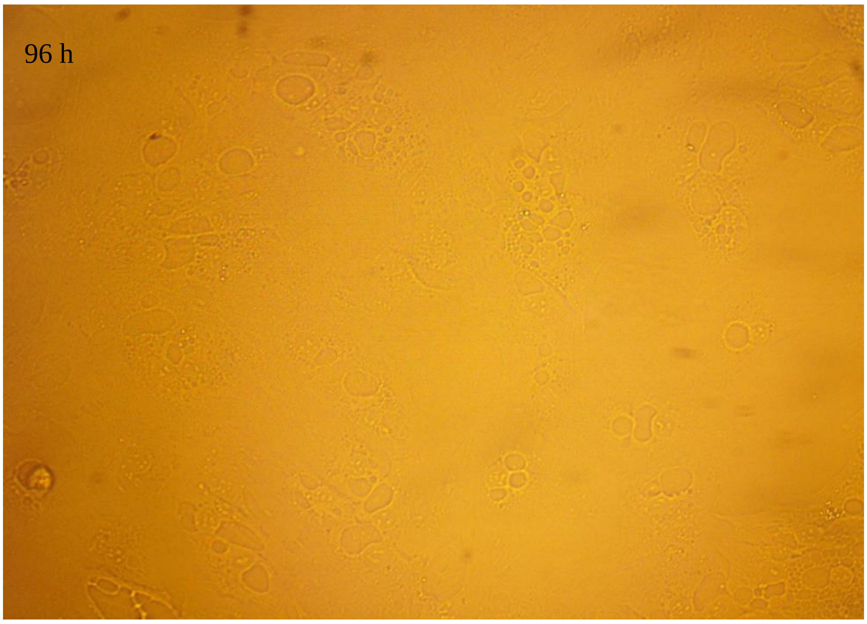

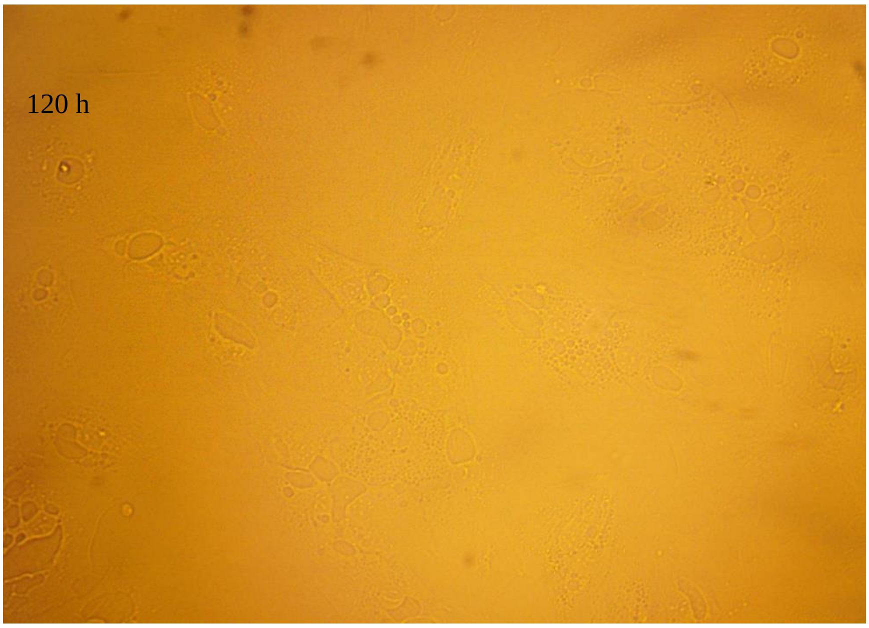

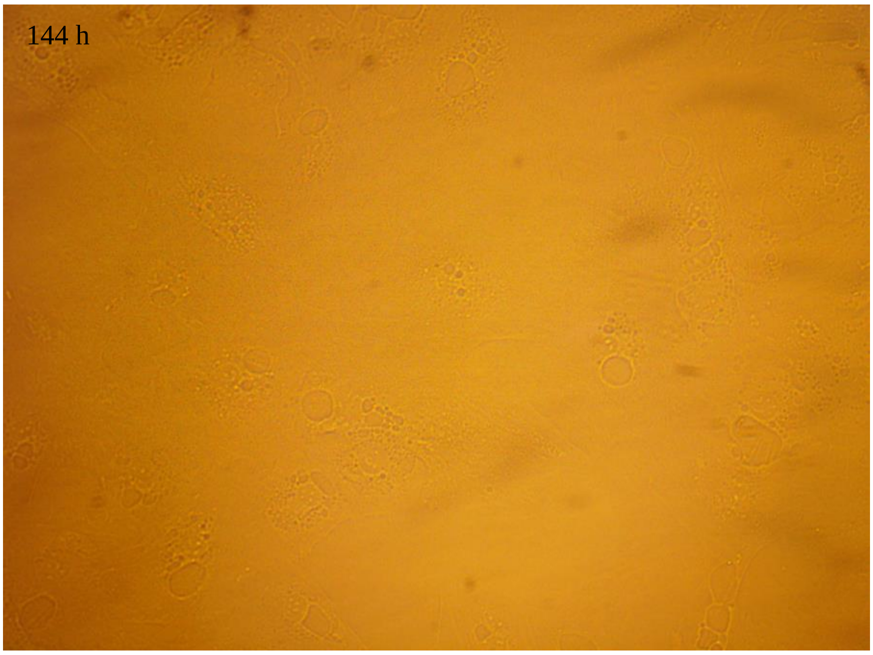
144 h

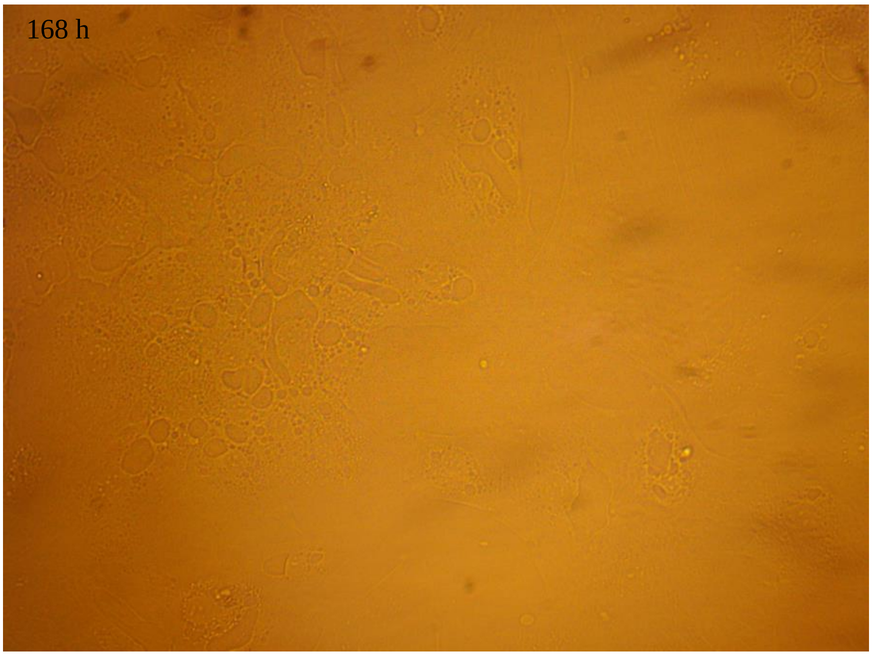
168 h

F4-F2 GCs:

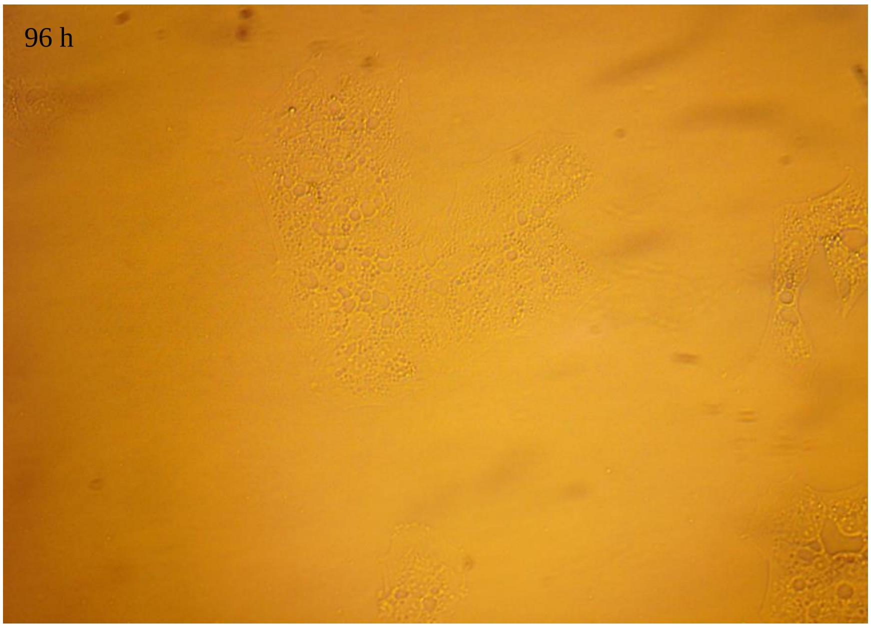

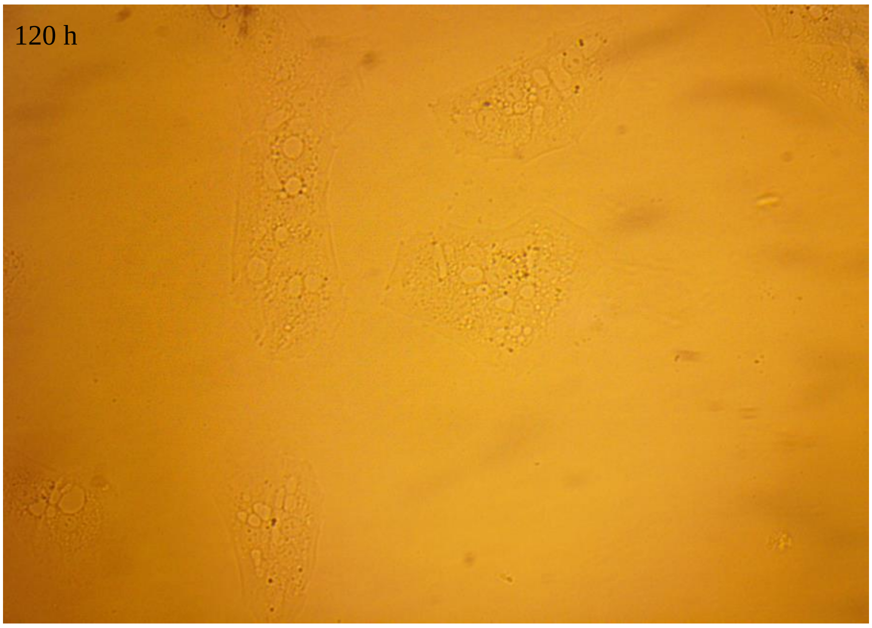

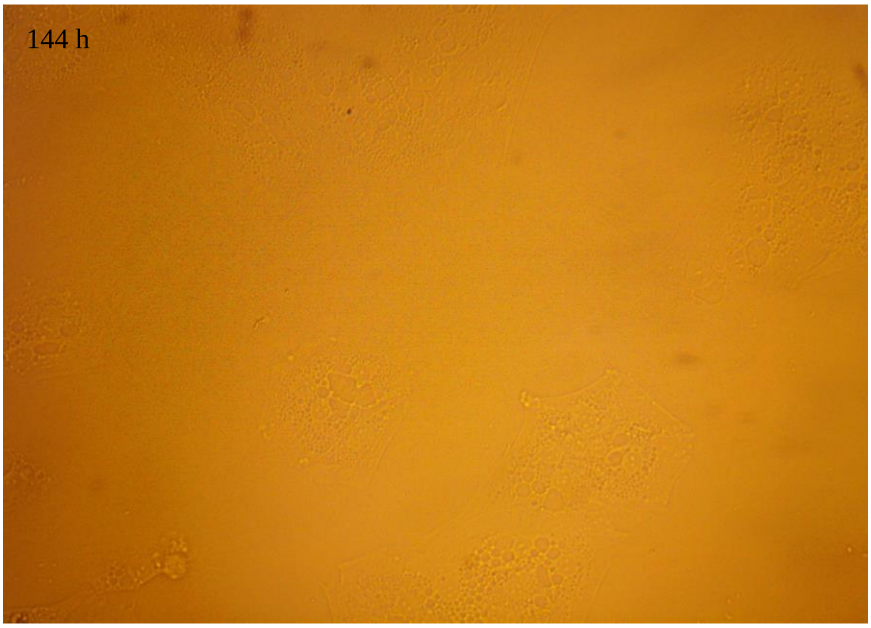
144 h

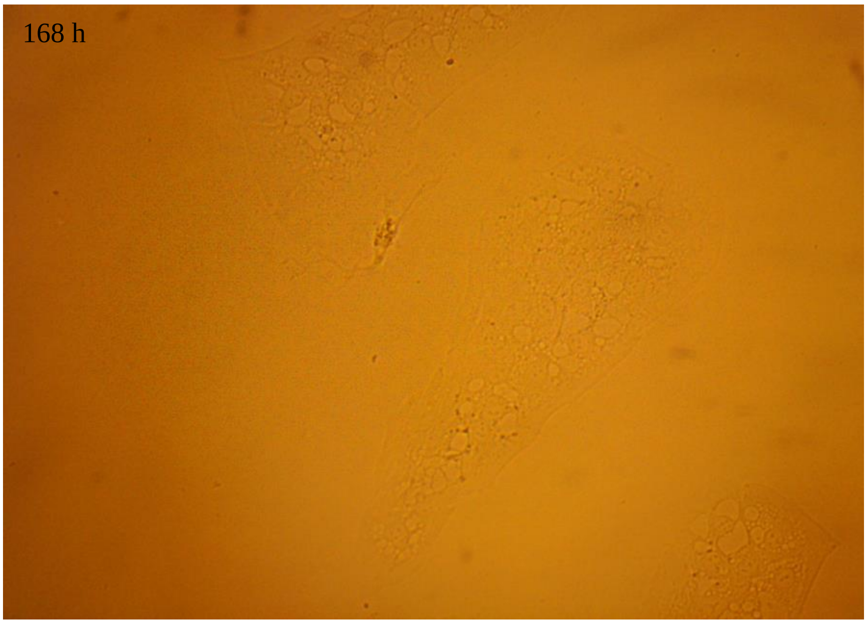
168 h

F1 GCs:

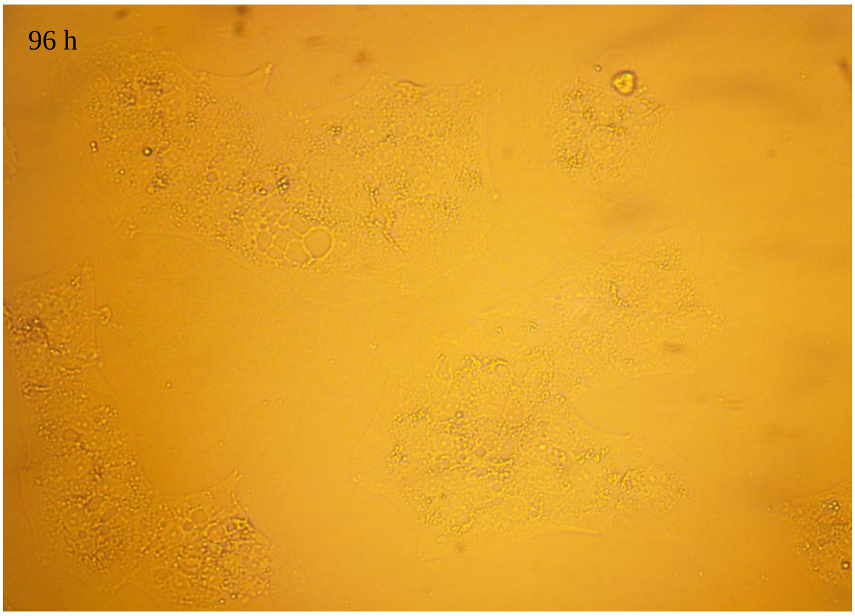

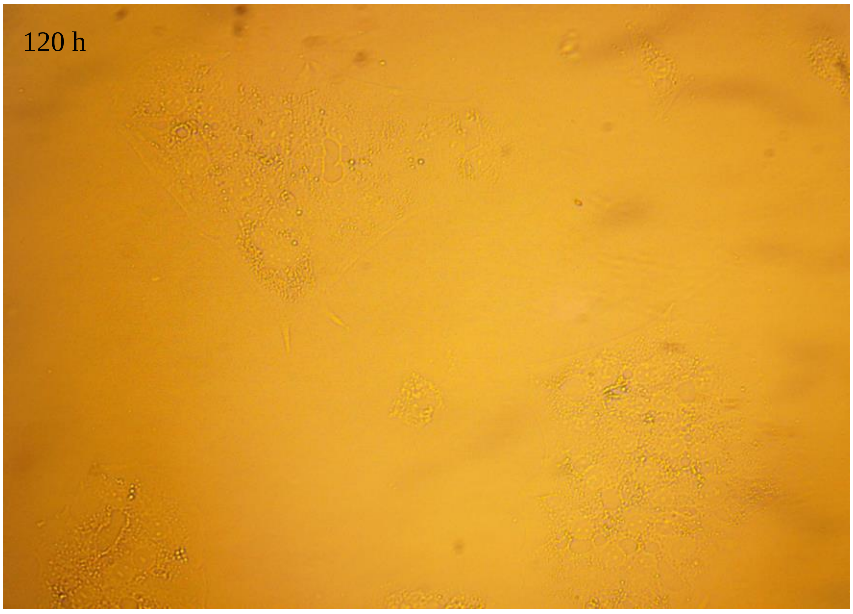

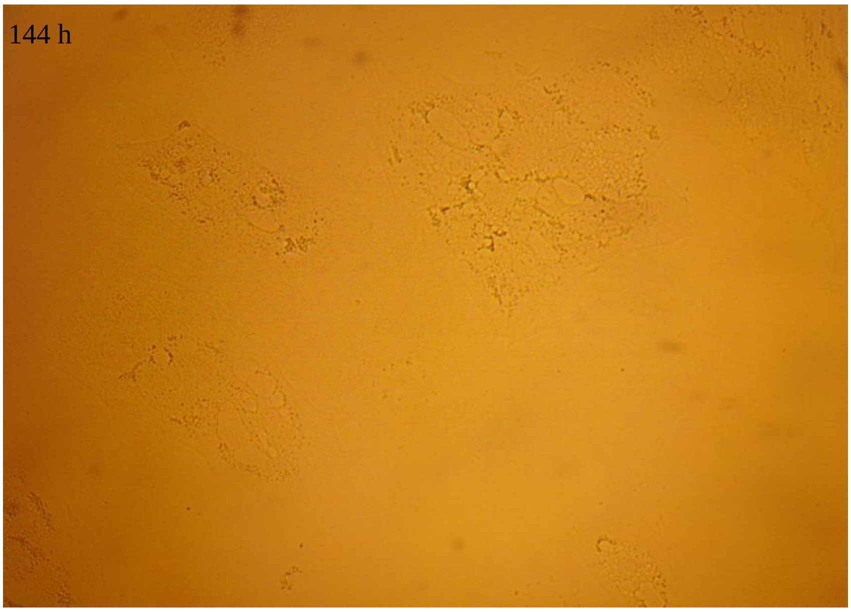
144 h

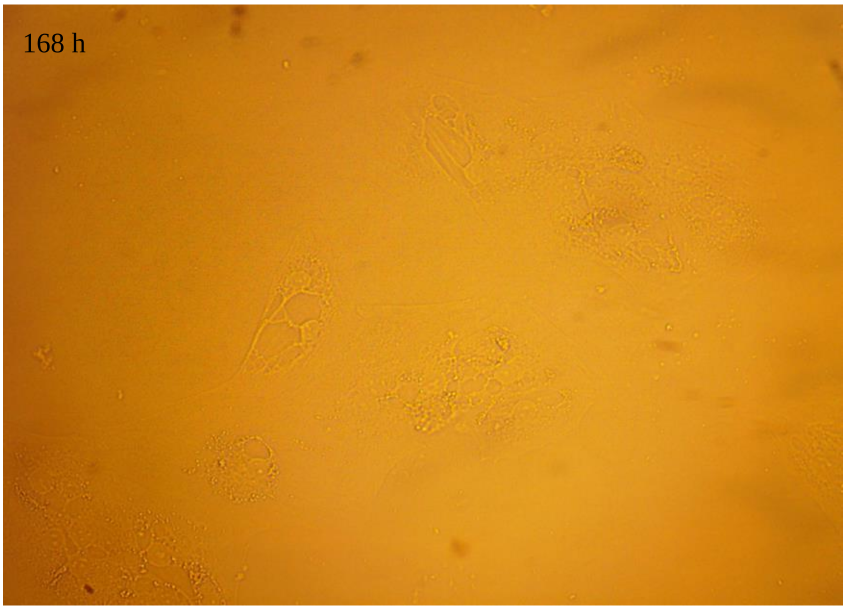
168 h
